# In vitro fluoride induced genotoxic effect on human blood lymphocyte cells and its amelioration by emblica officinalis extract

**DOI:** 10.1186/1755-8166-7-S1-P48

**Published:** 2014-01-21

**Authors:** Swati B Thakur, Mandava V Rao

**Affiliations:** 1 Human Genetic Unit, Department of Zoology, School of Sciences, Gujarat University, Ahmedabad, Gujarat, India

**Keywords:** antigenotoxicity, fluoride, amla, peripheral blood lymphocytes

## Background

Fluoride is a widespread industrial pollutant. Although, acute and chronic exposure of fluoride results in adverse health effects, in vitro studies demands for further evidences to conclude on the role of F as genotoxic agent. We have investigated the genotoxic properties of fluoride on peripheral blood lymphocyte cells and evaluated the protective effect of *Emblica officinalis* (Amla) against fluoride toxicity.

## Materials and methods

Peripheral blood lymphocytes were cultured and treated with different concentrations of fluoride (17 µM, 34 µM, and 51µM) and supplement with amla extract(20 µg) for the study of various genotoxic parameters such as sister chromatid exchanges (SCEs) and cytokinesis block micronucleus (CBMN) assay. To rule out the antioxidant properties of amla, indices like 1,1-diphenyl-2-picrylhydrazyl (DPPH) assay and High performance thin layer chromatography (HPTLC) were done.

## Results

Fluoride exhibited a significant increase in SCEs per metaphase plate (p<0.001) and SCEs per chromosome (p<0.05). Similarly, cell cycle proliferative index significantly decrease (p<0.001) in a dose-dependent manner in the three fluoride dose groups. Genotoxic indices such as nuclear deformities and frequency of micronucleus significantly (p<0.001) elevated with increased fluoride concentration. Furthermore, nuclear division index (NDI) and cell viability also noticed to be declined in fluoride treated cultures. Cultures with high dose of fluoride co-supplement with amla extract indicated a remarkable recovery in these genotoxic indices as comparable to control cultures. Antioxidant analysis of amla extract showed high free radical scavenging activity with EC_50_ value of 55.44 ± 0.12µg/ml.

## Conclusion

Amla has a strong antioxidant system to scavenge the free radicals generated through toxic effect. Amla showed an antigenotoxic effect against fluoride and thus has a great potential for the application in medicinal products.

